# Carbon fiber-reinforced PEEK as a framework material for single implant-retained mandibular overdentures

**DOI:** 10.1590/1807-3107bor-2025.vol39.018

**Published:** 2025-02-07

**Authors:** Guilherme Almeida BORGES, Marcelo Ferraz MESQUITA, Luana Figueiredo da Silva MATIAS, Thaís BARBIN, Leonardo Mendes Ribeiro MACHADO, Valentim Adelino Ricardo BARÃO

**Affiliations:** (a) Universidade Estadual de Campinas – Unicamp, Piracicaba Dental School, Department of Prosthodontics and Periodontology, Piracicaba, SP, Brazil.; (b) Renato Archer Center for Information Technology, Division of Technologies for Production and Health at the Campinas, SP, Brazil.

**Keywords:** Dental Implants, Finite Element Analysis, Dental Prosthesis, Dentures

## Abstract

This study aimed to assess the biomechanical behavior of reinforcement materials [cobalt chromium alloy (CoCr) and carbon fiber-reinforced polyether ether ketone (CFR-PEEK)] and their extensions (short: 15 mm and long: 25 mm) of single implant-retained mandibular overdentures (MO-1) by 3D finite element analysis (FEA), comparing them with MO-1 without a framework. Five models (CoCr-Short, CFR-PEEK-Short, CoCr-Long, CFR-PEEK-Long, and no framework) were created using the McNeel Rhinoceros 3D software, version 7.0. Stress distribution analysis by FEA was performed using the Optstruct solver, and a 30° oblique load (100 N) was applied in the anterior region (50 N on each central incisor). Biomechanical behavior was analyzed by overdenture displacement, maximum (MaxP) and minimum principal (MinP) stress, and von Mises stress. The MO-1 model ‘without a framework’ produced the largest dislocation, MinP stress on the mucosa, and MaxP stress in the cortical bone. Regardless of the extension of the framework, CFR-PEEK had minor MinP stress in the mucosa and lower overdenture displacement. The ‘CoCr-Long’ and ‘CFR-PEEK-Long’ models had higher von Mises stress on the implant housing, and MaxP stress on the O’ring nylon conventional implant and overdenture. The ‘CoCr-Short’ and ‘ CFR-PEEK-Short’ models showed a greater tendency for tension concentration solely in the framework. The incorporation of a framework into MO-1 decreased stress concentration on the overdenture, resulting in lower stress on the attachment, mucosa, implant, and peri-implant bone, irrespective of the material used. The short framework, however, performed better biomechanically in MO-1, and it is therefore the most recommended option. CFR-PEEK showed favorable biomechanical outcomes, and is suggested for reinforcement of MO-1.

## Introduction

Mandibular overdenture (MO) is often utilized for the rehabilitation of edentulous patients and, as observed in previous prospective studies, it is possible to perform it with a single (-1) implant.^1-4^ MO-1 is a low-cost, effective, and safe treatment option.^1-5^ A clinical trial using MO-1 demonstrated a 100% survival rate for implants in a 10-year follow-up.^1^ When compared to the two-implant MO, MO-1 offers a significant cost reduction.^5^ Additionally, it is a reasonable option for the rehabilitation of edentulous patients with limited mandibular bone volume.^2^ Although MO-1 is clinically reliable, it is prone to an increased incidence of fractures in the anterior region of the base, around the implant housing.^2,6,7^ Biomechanically, this failure begins with microcracks that propagate into cracks, eventually leading to complete failure of the prosthesis,^8^ thus increasing the need for prosthetic maintenance.^7^ Therefore, incorporating a framework into the MO-1 denture base could be a promising alternative to improve longevity and mechanical strength and reduce chair time for regular maintenance.^9,10^

Cobalt-chromium alloy (CoCr) is the most widely used material for manufacturing frameworks.^9,11-13^ Previous studies have confirmed that the reinforcement of MO-1 with a CoCr framework decreases stress in the anterior region of the prosthesis, thereby reducing the incidence of fractures.^9,11^ Although CoCr provides high flexural strength, stiffness, resilience, corrosion resistance, and low density,^12,13^ this metal alloy has some disadvantages, including fatigue failures under repeated loading, high weight, and increased incidence of allergic reactions.^12^ Therefore, given the possibility of ductile material failure, the von Mises equation plays a key role in the biomechanical assessment of MO-1.^14^

In a three-dimensional (3D) finite element analysis (FEA), it was observed that the CoCr framework enhanced the strength of the prosthesis base, without causing biomechanical limitations in adjacent structures such as the attachment, implant, and peri-implant bone.^9^ Another biomechanical study found that short framework-reinforced MO-1 had lower von Mises stress and total strain, suggesting higher material strength.^15^ However, conclusions on the ideal framework extension and the possibility of using different materials are still lacking. Considering these limitations of CoCr, recent studies have investigated polyether ether ketone (PEEK) as a promising material with the potential to improve clinical and biomechanical outcomes.^16^ This polymer presents low weight, excellent shock absorption properties and a Young’s modulus (Y) close to that of the cortical bone,^17^ allowing for the reduction of stress on the abutment and providing enhanced protection for a more uniform distribution of masticatory forces.^13,18-20^ The cortical bone around the dental implants experiences more stress than do other regions.^21,22^ In a FEA study assessing different framework materials, the carbon fiber-reinforced polyether ether ketone (CFR-PEEK) framework reduced cortical bone stress distribution around the implants in all designs of mandibular complete-arch implant restorations when compared with PEEK,^17^ exhibiting a higher Young’s modulus (Y) and improved stress distribution on both the implant and surrounding tissues compared to PEEK.^17,21^

FEA is the first step in testing new techniques and materials. It is a quick and efficient method for investigating stress on an MO-1 model and on adjacent structures such as the mucosa, cortical bone, and trabecular bone.^9,14^ Among in-silico methods, there is a consensus that stress distribution assessment by 3D FEA presents the most reliable simulation of a clinical scenario.^9,14,23^ Variables that may influence the clinical acceptability of CFR-PEEK include maximum (MaxP) and minimum principal (MinP) stresses, which predict stress distribution in peri-implant tissues,^18,24^ and such variables have not been investigated in current research.^14^ Overdenture displacement should also be investigated for understanding the different framework configurations of the MO-1 model.^23^ Therefore, this study aims to evaluate the biomechanical behavior of reinforcement materials [cobalt-chromium alloy (CoCr) and carbon fiber- reinforced polyether ether ketone (CFR-PEEK)] and their extensions (short: 15 mm and long: 25 mm) of single implant-retained mandibular overdentures (MO-1) by 3D FEA, when compared to MO-1 without a framework. The null hypothesis is that the stress distribution of reinforced MO-1 would not be affected by the selected framework material or extension.

## Methods

3D FEA models were designed in the pre-processing phase, based on the independent variables of this study (Table 1), considering the framework extension (short and long) and material composition (CoCr and CFR-PEEK). Solid elements (implant, ball attachment, implant housing, and O’ring nylon conventional implant) were included according to the manufacturer’s recommendations (Neodent, Curitiba, Brazil) and imported into Rhinoceros. A conventional implant model (3.75 mm diameter x 11 mm height, external hexagon) was used. The implant was placed in the midline symphysis of the mandibular residual ridge,^14^ regardless of the independent variable utilized in this study. The implant was considered to be fully osseointegrated at the bone–implant interface. Five 3D finite element models of the mandible were built in McNeel Rhinoceros 3D v7.0 software, assuming bilateral mandibular symmetry.

**Table 1:** Groups subjected to 3D finite element analysis.

Groups	Extension size	Framework material
CoCr-Short	Short-15 mm	CoCr
CFR-PEEK-Short		CFR-PEEK
CoCr-Long	Long-25 mm	CoCr
CFR-PEEK-Long		CFR-*PEEK*

Without a framework.

The geometry of the mandibular ridge was classified as type III (very rounded alveolar ridge with suitable height and thickness), as proposed by Cawood & Howell, considering both height (Y axis) and thickness (X axis) configurations.^25^ In the MO-1 model, the flange thickness was set at 2 mm, and at 10 mm for the MO-1 base. An offset with a standardized thickness of 2 mm was applied to the external bone surface that forms the cortical bone, which served as a reference for inferring trabecular bone thickness.^14,23^ The mucosal thickness was standardized at 2 mm.^25^

The framework was positioned 2 mm above the base of the implant housing, with perforations evenly distributed throughout the entire framework geometry to ensure proper clinical application and mechanical interlocking of the prosthetic material. The position of the framework in the posterior region varied according to its extension. In the short extension, the distal region of the framework corresponded to the distal occlusal region of the first premolar, whereas in the long extension, the distal region of the framework was located in the mesio-occlusal region of the second premolars. The mesh was generated using second-order tetrahedral geometry, with a node at each vertex and a node at the center of each edge, totaling 10 nodes per element. The geometries were exported as separated solids in standard tessellation language format (.stl) and then sequentially imported into Altair Hypermesh v.2022 software. Possible dimensional inconsistencies were verified before importing the .stl files and before the discretization process became infeasible.^14^ The material properties (Young’s modulus and Poisson’s ratio) are shown in Table 2. All the materials were assumed to be homogenous, linearly elastic, and isotropic. All conditions were set, and the analyses were carried out using HyperWorks 2022, OptiStruct (solver-c).

**Table 2:** Mechanical properties of materials used for 3D finite element analysis.

Component	Material	Young’s modulus (MPa)	Poisson ratio (v)
Implant, attachment, and implant housing	Titanium	103 400^23^	0.35^23^
Denture and artificial teeth	Acrylic resin	8 300^15^	0.28^15^
O’ring nylon	Nylon	2 400^38^	0.39^38^
Mucosa	Mucosa	34023	0.45^23^
Cortical bone	Cortical bone	13 700^26^	0.30^26^
Cancellous bone	Cancellous bone	1 370^26^	0.30^26^
Framework	CoCr	218 000^15^	0.33^15^
Framework	CFR-PEEK	15 000^17^	0.40^17^

Stress distribution was assessed by 3D FEA using the Optstruct solver, and an oblique load of 100 N at 30º was applied to the incisors (50 N per central incisor). The objective of this study was to simulate the average value of incisal force in edentulous patients rehabilitated with MO-1, mimicking a biomechanical scenario of protrusion.^9,26^ Once the experimental conditions were established, the models were analyzed using numerical equations (Ansys Workbench 11; Ansys Inc.) to simulate the mechanical responses of the bodies to loading. The boundary conditions related to movement and loading restrictions were determined according to the simplifications made by the Saint Venant principle,^27^ which involves crimping of the mandible, making the use of a complete mandible unnecessary, and also considers the plane that vertically crosses the condyle region. Contact conditions were set with the models fixed in the posterior condyle region. Sliding interfaces were assumed between the mucosa and prosthesis and between the component and O’ring nylon conventional implant, whereas the interfaces between the implant and the component and between the implant and the cortical bone were fixed. Freeze contacts were established for the other components.

MaxP stress was employed to predict tensile stress distribution in the cortical bone and possible material failure due to overload,^18,24,28-31^ using HyperView software. The same mathematical solver was applied to confirm the presence of compression in the mucosal tissue using MinP stress.^32-37^ MinP and MaxP stresses were applied to enable comparison of stress distribution between different framework materials. The von Mises equation was utilized for ductile components (CoCr framework, implants, and prosthetic components).^14,38^ Overdenture displacement was evaluated numerically, comparing different reinforced MO-1 models.^23^ The maximum Von Mises, MinP, and MaxP stresses were evaluated separately and plotted according to color codes (stress map). To enhance comprehension, color gradients were used to highlight the most critical points. The images obtained allowed for a visual comparison of the color scales and their gradients, in which warm colors represent higher stress values while cold ones indicate lower stress values.

## Results

**Figure 1:**
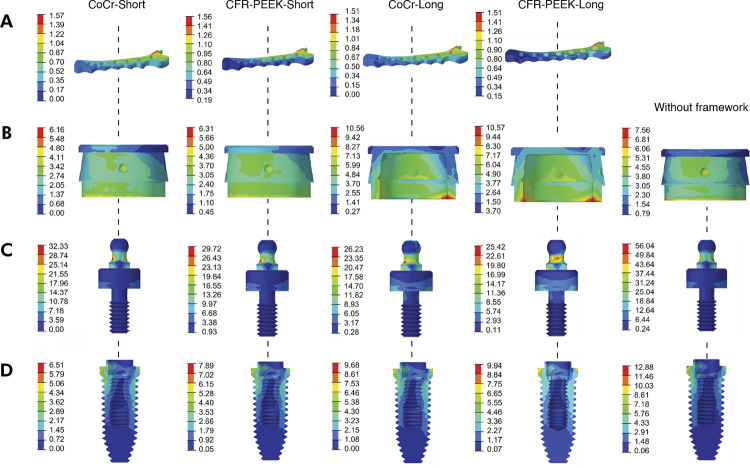
Von Mises stress distribution of ductile structures when an incisor was loaded with an oblique force of 100 N, and 30° of angulation. A, Framework. B, Implant housing. C, Attachment. D, Implant.

The von Mises stress values were measured in megapascal (MPa) and are also presented in Table 3, while the color-coded description can be seen in Figure 1. The highest von Mises stress value for the implant (12.88 Mpa) was observed in the MO-1 model ‘without a framework’, while the lowest value was seen in ‘CoCr-Short’, 6.51 MPa (49.46% of improvement) (Table 3). The model ‘without a framework’ also presented the highest von Mises stress for attachment (56.04 Mpa), however, the lowest value was generated in ‘CFR-PEEK-Long’, 25.42 MPa (54.64% of improvement) (Table 3). As for implant housing (Figure 1B), ‘CoCr-Long’ and ‘CFR-PEEK-Long’ yielded the highest stress values. Both groups showed similar values (10.56 MPa and 10.57 MPa respectively), increasing tension by 39.81% and 39.68% (Table 3). Conversely, the ‘CoCr-Short’ model had the lowest stress, 6.16 MPa (18.52% of improvement). All models showed similar values for framework (Table 3) - ‘CoCr-Short’: 1.57 MPa; ‘CFR-PEEK’: 1.56 MPa; ‘CoCr-Long’: 1.51 MPa; and CFR-PEEK: 1.51 MPa.

**Table 3:** Von Mises stress values (MPa) of ductile structures and relative improvement (%) of reinforced MO-1 compared with MO-1 ‘without a framework’.

Variable	Implant		Attachment		Implant Housing		Framework
	Stress value MPa	Improvement (%)	Stress value MPa	Improvement (%)	Stress value MPa	Improvement (%)	Stress value MPa
CoCr-Short	6.51	49.46	32.33	42.31	6.16	18.52	1.57
CFR-PEEK- Short	7.89	38.74	29.72	46.97	6.31	16.53	1.56*
CoCr- Long	9.68	24.84	26.23	53.19	10.56	-39.68	1.51
CFR-PEEK- Long	9.94	22.83	25.42	54.64	10.57	-39.81	1.51*
Without a framework	12.88	*	56.04	*	7.56	*	*

CoCr-Short: model with short extension and CoCr framework; CFR-PEEK-Short: model with short extension and CFR-PEEK framework; CoCr-Long: model with long extension and CoCr framework; CFR-PEEK-Long: model with long extension and CFR-PEEK framework. Without a framework, model without a framework. *data not applied.

**Figure 2:**
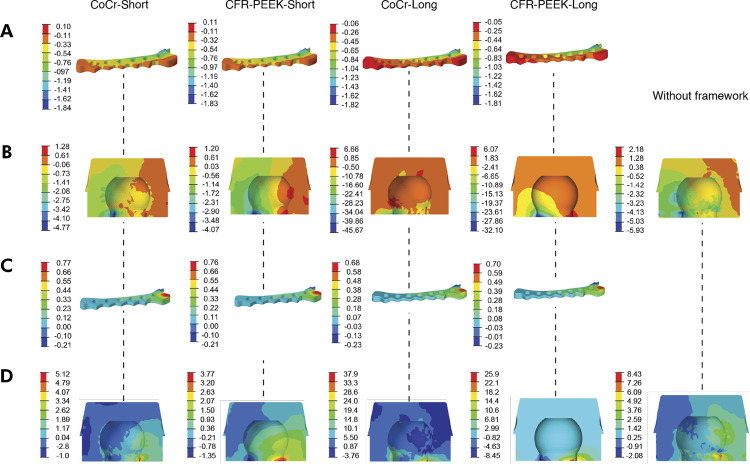
MinP (A and B) and MaxP (C and D) stress distribution when an incisor was loaded with an oblique force of 100 N, and 30º of angulation. A and C, Framework. B and D, O’ring nylon conventional implant.

When MinP was evaluated (Table 4), differences were observed among the groups (Figure 2). Regarding the mucosa, the model ‘without a framework’ presented the highest MinP stress (-0.46 MPa) and ‘CFR-PEEK-Short’ had the lowest value (-4.98 MPa) (improvement of 90.76%) (Table 4). However, the highest MinP stress value was recorded in the O’ring nylon (Figure 2B) conventional implant in the ‘CFR-PEEK-Short’ model (-4.07 MPa), with a 45.70% increase in tension ; on the other hand, the lowest value was observed in the ‘CoCr-Long’ model (-45.67 MPa) (87.02% of improvement) (Table 4). In terms of framework (Figure 2A), all models showed similar values. The ‘CoCr-Long’ and ‘CFR-PEEK-Long’ models yielded -1.817 MPa and -1.81 MPa, respectively, while the ‘CoCr-Short’ and ‘CFR-PEEK-Short models yielded -1.84 MPa and -1.83 MPa (Table 4). Nevertheless, when the overdenture was assessed, ‘CoCr-Short’ and ‘CFR-PEEK-Short’ presented higher MinP stress values (-92.46 MPa and -92.35 MPa), which increased by 27.99% and 27.84%, respectively. ‘CoCr-Long’ and ‘CFR-PEEK-Long’ presented the same MinP stress values (-118.9 MPa), regardless of the material used (0.59% of improvement) (Table 4).

**Table 4:** Minimum principal stress (compression stress) (MPa) values and relative improvement (%) of reinforced MO-1 compared with MO-1 ‘without a framework’.

Variable	Mucosa		O’ring nylon		Overdenture		Framework
	Stress value Mpa	Improvement (%)	Stress value Mpa	Improvement (%)	Stress value Mpa	Improvement (%)	Stress value Mpa
CoCr-Short	-1.04	55.77	-4.77	-24.32	-92.46	-27.84	-1.84
CFR-PEEK- Short	-4.98	90.76	-4.07	-45.70	-92.35	-27.99	-1.83
CoCr- Long	-0.69	33.33	-45.67	87.02	-118.9	0.59	-1.82
CFR-PEEK- Long	-0.71	35.21	-32.1	81.53	-118.9	0.59	-1.81
Without a framework	-0.46	*	-5.93	*	-118.2	*	*

CoCr-Short: model with short extension and CoCr framework; CFR-PEEK-Short: model with short extension and CFR-PEEK framework; CoCr-Long: model with long extension and CoCr framework; CFR-PEEK-Long: model with long extension and CFR-PEEK framework. Without a framework, model without a framework. *data not applied.

When MaxP stress was evaluated (Table 5) in the cortical bone, the highest stress occurred in the MO-1 model ‘without a framework’ (4.38 MPa). The inclusion of ‘CoCr-Short’ framework decreased the stress in the cortical bone (2.27 MPa) by 48.17%. As for the O’ring nylon (Figure 2D), the ‘long framework’ yielded higher MaxP stress values, with ‘CoCr-Long’ yielding 37.92 MPa, indicating a 349.82% increase; while ‘CFR-PEEK-Short’ had the lowest value (3.773 MPa) (55.28% of improvement). Similar MaxP values were observed for the framework (Figure 2C). The ‘CoCr-Short’ and ‘CFR-PEEK-Short’ models showed higher MaxP stress values (0.77 MPa and 0.76 MPa, respectively); while ‘CFR-PEEK-Long’ and ‘CoCr-Long’ models had lower MaxP stress values (0,70 MPa and 0,68 MPa). Thus, the material and extension of the framework do not present substantial differences in this case. When stress values were assessed in the overdenture, the ‘CFR-PEEK-Long’ and ‘CoCr-Long’ models showed larger tension (28.99 MPa and 28.97 MPa), increasing stress by 21.20% and 21.11%, respectively; while the ‘CoCr-Short’ and ‘CFR-PEEK-Short’ models yielded 18.85 MPa and 18.86 MPa, respectively, indicating 21.20% and 21.15% of improvement. The displacement values for each FEA model were measured in millimeters (mm). With respect to overdenture displacement, the model ‘without a framework’ exhibited the highest dislocation (0.05 mm in posterior region). The ‘CoCr-Short’ and ‘CFR-PEEK-Short’ models yielded 0.043 mm, and the smallest values were recorded for the ‘CoCr-Long’ and ‘CFR-PEEK-Long’ models (0.038 mm).

**Table 5:** Maximum principal stress (tensile stress) (MPa) values and relative improvement (%) of reinforced MO-1 compared with MO-1 ‘without a framework’.

Variable	Cortical Bone		O’ring nylon		Overdenture		Framework
	Stress value MPa	Improvement (%)	Stress value MPa	Improvement (%)	Stress value MPa	Improvement (%)	Stress value MPa
CoCr-Short	2.27	48.17	5.52	34.52	18.85	21.20	0.77
CFR-PEEK- Short	2.4	45.21	3.77	55.28	18.86	21.15	0.76
CoCr- Long	2.72	37.90	37.92	-349.82	28.97	-21.11	0.68
CFR-PEEK- Long	2.74	37.44	25.87	-206.88	28.99	-21.20	0.70
Without a framework	4.38	*	8.43	*	23.92	*	*

CoCr-Short: model with short extension and CoCr framework; CFR-PEEK-Short: model with short extension and CFR-PEEK framework; CoCr-Long: model with long extension and CoCr framework; CFR-PEEK-Long: model with long extension and CFR-PEEK framework. Without a framework, model without a framework. *data not applied.

## Discussion

Reinforced MO has been suggested to prevent fractures, which are the most frequent prosthodontic complication in MO-1 treatment.^3^ In this study, the null hypothesis was rejected because the reinforced MO-1 model exhibited different biomechanical behaviors. Reinforced MO-1 showed lower stress on the denture, attachment, implant, and peri-implant bone, regardless of the type of material. Absence of a framework clearly shows that the MO-1 model is more susceptible to failure because it accumulates more tension. A prospective cohort study with a follow-up of 12 to 80 months showed that the overall incidence of fractures in MO-1 was 32.2%.^6^ Midline denture fracture was a common complication.^6^ The inclusion of a framework can extend the lifespan of MO-1.^9-11^ Reinforced MO-1 decreases stress concentration around the implant.^9^ This reduction could avoid denture base fracture in the anterior region.^9,10,15^ Frameworks are assumed to act as stress collectors because of their biomechanical properties^9,10^ such as resilience, stiffness, and high flexural strength.^12,13^ A 3D FEA study found that a short framework presented lower von Mises stress and total deformation, indicating an increase in implant longevity.^15^

Differences were observed in von Mises stress values (Table 3). More stress was generated on the implant housing of reinforced MO-1 with a long framework. The highest stress for the attachment was observed in the MO-1 model ‘without a framework’. The framework seems to protect the implant and the attachments, regardless of framework extension or material. The attachment was the first component to fail under masticatory loads, which is corroborated by other studies suggesting that the attachment system is an important risk factor for mechanical complications of MO.^23^ Stress distribution on the framework yielded similar results in both types of frameworks.

Young modulus (Y) of the framework material could explain the differences in stress distribution.^9^ The CoCr material, which has a high Y value, tends to concentrate the stress from the loads.^9^ On the other hand, materials with lower Y values, such as CFR-PEEK, tend to transfer stress to adjacent materials with higher Y values. However, CFR-PEEK presents a Y value close to that of the mandibular bone, allowing for reduction of stress transfer to the abutment, and increasing protection because of more evenly distributed masticatory forces.^13,18^ Although CFR-PEEK and CoCr presented different Y values under the simulated clinical conditions, both materials showed a similar biomechanical behavior.

MinP stress decreased (Table 4)^24,28,32-37^ with the inclusion of a framework, which protected the mucosa, especially in the ‘CFR-PEEK-short’ model. The ‘long framework’ provided better protection for the O’ring nylon conventional implant, independently of the material. The MO-1 model with a ‘long framework’ or ‘without a framework’ provided less MinP stress in comparison to the ‘short framework’. Besides, higher MinP stress was observed on the overdenture, with short frameworks showing similar MinP stress values, regardless of the material or extension. When MaxP stress on the cortical bone was assessed (Table 5),^18,24,28-31^ higher stress occurred in the MO-1 model ‘without a framework’. The highest stress during implant loading is transferred to the first contact area. Thus, the cortical bone around the neck and the bottom part of the dental implant concentrated more stress than the other regions.^21,22^ The ‘short framework’ showed a greater tendency to concentrate tension, offering more protection to the O’ring nylon conventional implant and to the overdenture, irrespective of the material used. Upon loading, the denture base sank into the mucosa, and the anteroposterior MO displacement caused the implant to intrude into the bone, resulting in compressive stress on the mucosa in the anterior region.^23^ Thus, greater MO-1 displacement may lead to greater MinP stress on the mucosa. In this study, the MO-1 model ‘without a framework’ produced a slightly higher dislocation and MinP stress on the mucosa. Nevertheless, these findings are not expected to have a clinically significant effect. The ‘long framework’ generated the smallest dislocation probably due to the relationship between the weight of the framework and MO displacement. Therefore, further biomechanical research comparing the weight of the framework and MO displacement is needed.

The current findings of stress distribution further emphasize the importance of incorporating a framework into MO-1 to reduce stress, preventing fatigue on the O’ring nylon conventional implant and improving biomechanical behavior. The risk of fatigue in the implant housing region increases the risk of O’ring nylon conventional implant displacement during prolonged mastication. This compromises the stability of the MO-1 and can lead to mechanical failures such as deformations or fractures.^14,23^ This finding may be related to the resilient O’ring nylon conventional implant surrounding the ball system, acting as a stress breaker, and increasing the flexibility of the system due to the elastic properties of the O’ring.^14^ While MO-1 is an affordable, effective, and safe treatment option,^5^ the inclusion of a framework in MO-1 adds to the cost. However, the framework can help decrease the most common maintenance event, which is the replacement or adjustment of the retentive elements, thereby reducing long-term follow-up costs, particularly those associated with frequent dental recall appointments.^4,5^ It is paramount to highlight that the MO-1 model is not as effective in the long term as two-implant MOs or when compared to the fixed protocol prosthesis. There are, however, specific cases in which MO-1 could be the most suitable choice,^40,41,42^ such as rehabilitation of edentulous patients with atrophic mandibular ridge, when it is not possible to plan more dental implants^1,2^ or even when rehabilitation with a conventional complete denture (CD) would not have a favorable prognosis. Moreover, studies describe the short and long-term effectiveness and improvement in the outcomes of patients using MO-1^3,5,40^ when compared to CD, as MO-1 is considered a well-tolerated clinical solution with no safety concerns.^40^

Even though FEA is widely used to assess biomechanical behavior,^12,14^ this study had some limitations, also pointed out in previous studies.^14,15,23,27,38^ The methodology did not accurately simulate the dynamic loading associated with mastication, nor did it did attempt to replicate the complex biomechanical environment of the oral cavity,^17^ representing only an initial approach to evaluating the use of a framework.^9^ The analysis allowed generating initial computational data for the identification of potential clinical implications and new treatments. Future studies should focus on the development of new solutions to reduce the incidence of fractures in MO-1.^9^ An ideal clinical condition of the mandibular ridge was simulated,^25^ with a vertically oriented implant and a physiological oblique load on the mandibular central incisors. This approach is justified because it mimics the food-cutting movements of edentulous patients using MO-1.^9,26^

Given that the highest incidence of fracture on MO-1 occurs in the anterior region,^2,6,8^ a pilot study was previously performed to assess the stress distribution in reinforced MO-1. This pilot study revealed that the application of posterior load does not contribute significantly to stress in the anterior region. While CFR-PEEK has good mechanical properties,^17,38,39^ its use still requires careful discussion and well-designed clinical studies to evaluate prosthetic maintenance and its cost-effectiveness.^14,23^

## Conclusion

Based on the findings of this study, the following conclusions were drawn:

- The ‘short framework’ decreased MaxP stress on the overdenture (up to 21.20%), on the O’ring nylon conventional implant (up to 55.28%), and on the peri-implant bone (up to 48.17%). It also reduced MinP stress on the mucosa (up to 90.76%), and von Mises stress on the implant (up to 40.46%), attachment (up to 46.97%), and implant housing (up to 18.53%).
- The short extension showed a greater tendency to concentrate tension only on the framework, and it is therefore the most recommended option for reinforced MO-1.
- ‘CFR-PEEK’ yielded the lowest MinP stress on the mucosa (90.76%), showing a good biomechanical behavior, suggesting its potential clinical use in MO-1.
