# Erratum

**DOI:** 10.1590/1807-3107bor-2025.vol39.018err

**Published:** 2025-03-31

In article Carbon fiber-reinforced PEEK as a framework material for single implant-retained mandibular overdentures, with DOI number https://doi.org/10.1590/1807-3107bor-2025.vol39.018, published in the journal Brazilian Oral Research, v. 39, e018, 2025:

on p. 1

**The correct order of authors is:**

Luana Figueiredo da Silva MATIAS^(a)^

Thaís BARBIN^(a)^

Leonardo Mendes Ribeiro MACHADO^(b)^

Valentim Adelino Ricardo BARÃO^(a)^

Marcelo Ferraz MESQUITA^(a)^

Guilherme Almeida BORGES^(a)^

In the captions of the p. 3, 5, 7, 9 and 11

**Where is read:**

*Borges GA, Mesquita MF, Marias LFS, Barbin T, Machado LMR, Barão VAR*

**Read:**

*Matias LFS, Barbin T, Machado LMR, Barão VAR, Mesquita MF, Borges GA*

